# Health Disparities in Hepatitis D Virus Management: A Case Series on Improving Patient Access to Bulevirtide Therapy

**DOI:** 10.1016/j.gastha.2026.101091

**Published:** 2026-08-07

**Authors:** Ahmed Elsharkawy, Lindsay Horobin, Rhys Oakley, Sital Shah

**Affiliations:** 1 Liver, Queen Elizabeth Hospital Birmingham, University Hospitals Birmingham NHS Foundation Trust, Birmingham, UK; 2 Hepatology Department, Barnsley Hospital NHS Foundation Trust, Barnsley, UK; 3 Infectious Diseases, University Hospital of Wales, Cardiff, UK; 4 Pharmacy Department and Institute of Liver Studies, King's College Hospital NHS Foundation Trust, London, UK; 5 Faculty of Life Sciences and Medicine, King's College London, London, UK

**Keywords:** Hepatitis D Virus (HDV), Bulevirtide, Health Disparities, Treatment Access

## Abstract

**Background and Aims:**

Migrants and those from marginalized backgrounds with hepatitis D virus (HDV) often face social or health challenges that can impair access to care and adherence to treatment. This case series seeks to highlight that such challenges can be overcome and should not be seen as insurmountable barriers to the initiation of bulevirtide for HDV.

**Methods:**

Four consenting, anonymized patients were retrospectively identified from UK centers by a multidisciplinary authorship group.

**Results:**

Identified patients represented 4 different social circumstances that may impair treatment adherence: patient 1 had a history of mental health issues and was recently evicted; patient 2 was living in a homeless shelter, without access to a fridge or to a safe, confidential space for bulevirtide administration and disposal; patient 3 had a history of illicit drug use and previously failed to engage with care; and patient 4 failed to maintain injections following an unplanned trip to Africa. Each patient achieved positive results with bulevirtide therapy—demonstrating a reduced viral load and improvements in key measures of hepatic function—with adjustments made to their care to account for their unique needs. This included initiating therapy at a time that accommodated their personal circumstances, pausing treatment as needed, and working with third-party services to provide practical support that could address medication storage and logistical challenges.

**Conclusion:**

By shifting the perception that social circumstances are barriers to adherence, healthcare providers can promote a more inclusive approach to initiating, maintaining, and, where necessary, restarting bulevirtide therapy, ultimately improving health outcomes for patients with HDV.

## Introduction

Hepatitis D virus (HDV) is a defective subvirus that requires the presence of the hepatitis B virus (HBV) to propagate, as it cannot establish an infection on its own.^1^, ^2^, ^3^ HDV/HBV coinfection is the most severe form of viral hepatitis and is associated with more severe liver disease, more rapid progression to cirrhosis, and increased risk of hepatic decompensation and death vs HBV infection alone.^2^, ^3^, ^4^, ^5^

HDV is estimated to affect 12–15 million people globally,^1^^,^^5^^,^^6^ yet prevalence estimates are incomplete, and there is drastic geographic variation.^1^^,^^6^ In Europe, prevalence has declined over recent decades, though this decline has slowed in line with rising migration from low- or middle-economic countries where HDV infection is endemic.^1^^,^^7^ Full prevalence is likely underestimated, as limitations of diagnostics and poor access to healthcare in HDV-endemic countries mean current practices fail to identify all infected individuals.^1^ A definitive diagnosis of HDV requires the identification of HDV ribonucleic acid (RNA);^1^^,^^8^ however, access to HDV RNA testing is limited worldwide, and tests are often insufficiently standardized or validated.^1^^,^^6^ As such, many epidemiological studies are anti-HDV antibody-based and are limited by the lack of RNA data available for analysis.^5^^,^^6^

Effective HDV therapies are available in some countries.^9^^,^^10^ Despite lacking an indication for HDV, pegylated interferon (PEG-IFN) is widely used as an HDV treatment and is recommended for HDV and compensated liver disease.^8^^,^^9^ As of May 2026, bulevirtide is the only licensed therapy for chronic HDV infection, indicated in the European Union and the United Kingdom for the treatment of chronic HDV infection in plasma (or serum) HDV-RNA-positive adult and pediatric patients with compensated liver disease.^9^^,^^11^^,^^12^ No therapies are currently indicated for the treatment of HDV in the United States.^9^

In clinical trials, bulevirtide has been shown to reduce HDV RNA and alanine aminotransferase (ALT) levels in patients with chronic HDV infection.^13^ However, in real-world practice, daily subcutaneous administration and specific storage requirements for bulevirtide^11^ can present adherence challenges for patients.^7^

In addition, many individuals with HDV face social or health-related challenges that can potentially impede their ability to adhere to treatment. Mental health struggles, such as feelings of stress or helplessness regarding their diagnosis, can impair medication adherence.^14^ Equally, patients with HDV can face frequent stigma and discrimination,^15^ with fear of judgment restricting engagement with treatment as individuals may not wish to inadvertently disclose their infection status by administering or storing treatment where it may be seen by others.^14^ Given the high prevalence of HDV in individuals from migrant populations, language barriers, unstable financial situations, and low education or health literacy can also be particular challenges for this patient group,^7^^,^^16^ and each has been linked to poor adherence rates.^14^^,^^17^

Assumptions about adherence can be made in clinical practice, with healthcare professional (HCP) biases potentially leading to impaired treatment access for patients based on their perceived ability to adhere to treatment.^18^ There are notable gaps in the literature regarding the impact of such assumptions on prescribing practices for HDV. However, for hepatitis C virus, research has demonstrated that preconceived notions on an individual’s ability to adhere to treatment can impact HCP prescribing practices, with one study noting that 38% of prescribers were “rarely” or “never” willing to treat patients with one or more barrier to treatment adherence.^19^

This case series aims to highlight that certain social circumstances are not insurmountable barriers to effective HDV therapy and demonstrate that, with appropriate support, such circumstances can be overcome to help patients with HDV achieve positive results with bulevirtide therapy.

## Methods

Four cases of patients with HDV infection were retrospectively identified by a multidisciplinary authorship group from centers across the United Kingdom. The authorship team comprised a consultant hepatologist (A.E.), a clinical nurse specialist (L.H.), and 2 specialist clinical pharmacists (R.O. and S.S.). Each author selected a patient from their respective center, with patients chosen to reflect a range of backgrounds and challenging social scenarios. All patient details were anonymized to protect patient confidentiality, and informed consent was obtained from each patient prior to inclusion. Any adverse events experienced by the patients were reported to the Gilead Sciences Ltd Drug Safety team.

## Results

### Patient 1

A 47-year-old female patient from Moldova was referred to pharmacy services at King’s College Hospital National Health Service (NHS) Foundation Trust for access to bulevirtide therapy. The patient had first been admitted to a hospital in her home country aged 13 years, presenting with liver dysfunction that was later believed to be HBV with HDV superinfection. She first received treatment for HDV in 2013, achieving end-of-treatment response with interferon therapy; however, she experienced a relapse after discontinuing treatment in 2014, and a history of severe seasonal depression and anxiety made her ineligible for further courses of PEG-IFN. At the time of King’s College Hospital NHS Foundation Trust referral, her current medication included the antiviral entecavir (0.5 mg once daily [OD]), as well as the antidepressants sertraline (200 mg OD) and amitriptyline (10 mg at night).

The patient presented to the pharmacy team experiencing a severe depressive episode, largely driven by concerns regarding her HDV diagnosis. Despite this, the patient was looking forward to starting bulevirtide therapy. She came to the clinic 1 month later to start treatment, whereby her mental health had improved, and she was ready to commence therapy. Magnetic resonance elastography showed F2 fibrosis, making her eligible for bulevirtide (2 mg) therapy per UK reimbursement criteria.^12^

Two weeks into treatment, the patient’s ALT improved (Table 1); however, she had been evicted and was forced to relocate. As a result, the patient was stressed and struggled with the routine of daily injections. A mutual decision was made to pause HDV treatment until her housing situation resolved and her mental health improved. Discontinuation of bulevirtide therapy can lead to the reactivation and exacerbation of infection; therefore, careful monitoring of liver function tests, as well as HBV deoxyribonucleic acid and HDV RNA viral load, is recommended.^11^ As such, follow-up was planned for once every 3 months, and the patient was asked to reach out once ready to restart therapy. Unfortunately, the patient experienced a disease flare upon treatment discontinuation.

**Table 1:** Blood Values During Bulevirtide Therapy: Patient 1

	HDV RNA (IU/mL)	ALT (U/L)
Baseline prior to treatment initiation	4274	250
Bulevirtide treatment initiated		
Treatment week 2	-	50
Bulevirtide treatment paused		
1 month after stopping	9332^a^	421^a^
1.5 months after stopping	4333	192
Bulevirtide treatment restarted		
When restarting	7568	71
Treatment restart week 4	6297	32

a Flare after stopping treatment.

The patient’s housing situation was resolved 5 months later, but her new home lacked a fridge. She was keen to restart therapy but was concerned about the lack of appropriate storage. Following a discussion between the pharmacist and the consultant, the patient restarted bulevirtide; treatment was provided month-by-month rather than in the usual 3-month supply to account for her storage challenges.

One month after restarting bulevirtide therapy, the patient’s ALT levels returned to normal range (Table 1). At the same time, she gained access to a fridge and could continue therapy with appropriate storage. On restarting treatment, the patient received follow-up telephone appointments every 2 weeks for 2 months. This was subsequently reduced to once every 3 months, after which the patient was encouraged to reach out to the team if she had any concerns.

### Patient 2

A 28-year-old Black African male was screened for blood-borne viruses and other infections by a health inclusion service. Originally from South Sudan, the patient had been in the United Kingdom for 5 months prior to first presentation, having arrived alone, seeking asylum. He had a history of depression for which he was not receiving treatment, was on no regular medication, had no known drug allergies, and did not drink alcohol.

The patient was screened for HBV and HDV in the Infectious Diseases Outpatient Clinic at University Hospital of Wales. The patient was found to be anti-HDV reactive, though HDV RNA was not detected, and therefore this was perceived to be due to a previous HDV infection. The patient was seen in a tuberculosis (TB) clinic 2 months later and started treatment for latent TB infection. Abnormal ALT results were observed at this time point, which were thought to be a result of latent TB infection therapy. Thirteen months after the initial screening, repeat testing identified HDV RNA, and the patient was diagnosed with HBV/HDV coinfection. As the patient was ineligible for PEG-IFN therapy due to mental health concerns, the decision was made to wait for the UK approval of bulevirtide.

Bulevirtide was approved for reimbursement by the National Institute for Health and Care Excellence in June 2023;^12^ however, the patient’s living situation presented significant challenges for treatment. The patient was sleeping in a homeless shelter, which he could only access between 20:00 and 08:30, and he did not have access to a fridge or a safe, confidential space for bulevirtide administration and disposal. Consequently, the patient was referred to the Outpatient Parenteral Antimicrobial Therapy (OPAT) team, a service that provides the delivery of antimicrobial medication outside a hospital setting.^20^ The OPAT service operates 365 days a year, enabling prompt access to treatment, storage per the license, education, and confidentiality (RO’s personal experience). The patient initiated bulevirtide (2 mg) with support from OPAT and had an excellent response to therapy (Table 2).

**Table 2:** Blood Values During Bulevirtide Therapy: Patient 2

	HBV DNA (IU/mL)	HDV RNA (IU/mL)	ALT (U/L)
Baseline prior to treatment initiation	<10	660,000	34
Bulevirtide treatment initiated			
Treatment week 2	-	240,000	36
Treatment week 6	Not detected	-	-
Treatment week 10	-	3300	25
Treatment month 9	<10	<250	23
Treatment month 12	71	810	267

DNA, deoxyribonucleic acid.

### Patient 3

A 46-year-old Latvian male patient was first diagnosed with HBV in July 2018 via a blood-borne virus screening service at Barnsley Hospital. The patient was later diagnosed with HBV/HDV coinfection in April 2019 as part of routine screening. The patient had a history of intravenous drug use; however, the most likely source of infection was deemed to be a medical procedure in Latvia in 2016.

Between 2018 and 2020, the patient failed to attend multiple appointments with the hepatitis team, including abdominal ultrasound scans and FibroScans. At that time, he was receiving opioid substitution therapy in drug services; therefore, in January 2020, appointments with the hepatitis team were initiated in the drug services clinic to reduce appointment burden and promote engagement with his HDV management. Unfortunately, the COVID-19 pandemic and lockdown began shortly afterward, and the patient disengaged. Contact via drug services was reinitiated in November 2020. Following counseling, the patient started weekly PEG-IFN injections and 6-monthly hepatocellular carcinoma surveillance.

The patient remained engaged with treatment until February 2022, completing 46 weeks of PEG-IFN therapy, but then discontinued due to eyesight deterioration. The patient then became uncontactable and did not attend multiple follow-up appointments. He was eventually seen again in drug services in November 2022 and completed clinical assessments but disengaged shortly after. The patient was next seen in drug services in December 2023. Abdominal ultrasound showed coarse liver echotexture and a spleen at the upper limit of normal.

He attended a hepatology outpatient appointment in April 2024 (Table 3), following which the hepatitis multidisciplinary team agreed to start entecavir 0.5 mg tablets OD; outpatient treatment was initiated in July 2024. The patient continued to take entecavir without any reported side effects. In August 2024 he received counseling on bulevirtide therapy, commencing treatment in September 2024.

**Table 3:** Blood Values During HDV Therapy: Patient 3

	HBV viral load (IU/mL)	HDV RNA (IU/mL)	ALT(U/L)	Bile acids (μmol/L)
Baseline prior to HDV treatment initiation	264	16,000,000	52	-
Entecavir treatment initiated				
Treatment month 1	0	-	39	-
Bulevirtide treatment initiated				
Treatment month 1	0	-	42	-
Treatment month 2	-	-	37	-
Treatment month 5	0	669,000	38	2

Bulevirtide administration training was provided by the Hepatology Nurse Specialist, with treatment delivered to the patient via the Homecare Medicines Service. Administration technique was reviewed daily, either at the hospital or in drug services, until the patient was deemed competent and felt comfortable. By November 2024, he was administering bulevirtide injections daily but was experiencing pain at the injection sites. Injection technique was reviewed, and he was encouraged to alternate injection sites. As of February 2025, the patient continued to inject daily with no further known issues (Table 3).

### Patient 4

A 40-year-old male was referred to the hepatology team at the Queen Elizabeth Hospital Birmingham with persistent elevated ALT and aspartate aminotransferase. He had been diagnosed with HBV 7 years previously, receiving PEG-IFN therapy and ursodeoxycholic acid for an unknown duration. In the year prior to his referral, a biopsy conducted at his local center had shown bridging fibrosis and moderate chronic inflammatory activity; based on his elevated immunoglobulin G (24.4 g/L) it was assumed he had autoimmune hepatitis and was thus treated with tenofovir 245 mg OD, azathioprine 50 mg OD, and prednisolone 10 mg OD. For the first time since his HBV diagnosis he was tested for HDV, which showed him to be HDV immunoglobulin G-positive with a viral load of 7,900,000 IU/mL (HBV deoxyribonucleic acid 22 IU/mL). He was formally diagnosed with HBV/HDV coinfection and was gradually weaned off immunosuppression over the next 3 months.

A repeat biopsy showed compensated F4 fibrosis and endoscopy showed portal hypertensive gastropathy. Hepatocellular carcinoma surveillance was initiated, as well as PEG-IFN therapy, to which he had an excellent virological response; after 5 months of therapy his HDV viral load was undetectable, though blood monitoring became difficult due to the COVID-19 pandemic. Four months after completing the 48-week course of therapy, repeat testing confirmed HDV negativity and normal ALT and aspartate aminotransferase levels. Seven months thereafter there was evidence of viral recurrence; however, at the time, he had well-compensated disease and, following discussion at the viral hepatitis multidisciplinary team meeting, a decision was made to wait for UK approval of bulevirtide.

In September 2022, due to the presence of Child–Pugh grade A cirrhosis, the patient was eligible for bulevirtide through an early access program and started treatment (2 mg), initially achieving a good virological and biochemical response (Table 4). However, there were some concerns about the patient’s injection technique, and he reported some muscle aches.

**Table 4:** Blood Values During Two Attempts at Bulevirtide Therapy: Patient 4

Week	HDV RNA (IU/mL)	ALT (U/L)	AST (U/L)	Albumin, (g/L)	Bile salts, (μmol/L)
Baseline prior to HDV treatment initiation	620,000	168	112	33	35
Bulevirtide treatment initiated (first attempt)					
Treatment week 4	43,000	159	97	38	11
Treatment week 10	100,000	75	53	37	49
Treatment week 13	-	106	70	34	85
Treatment week 17	770,000	191	122	36	-
Treatment week 21	-	251	142	36	-
Bulevirtide treatment paused					
Post discontinuation week 2	470,000	144	96	35	11
Post discontinuation week 9	-	187	118	35	11
Baseline prior to restarting treatment	240,000	149	-	-	-
Bulevirtide treatment initiated (second attempt)					
Treatment week 2	110,000	183	-	-	41
Treatment week 4	64,000	147	-	-	-
Treatment week 7	51,000	99	-	-	-
Treatment week 12	18,000	61	-	-	36
Treatment week 24	1300	46	-	-	106
Treatment week 41	2100	44	-	-	-
Treatment week 54	380	29	-	-	-

AST, aspartate aminotransferase.

The improvement in viral measures reversed between weeks 10 and 17 when the patient took an unplanned extended trip to Africa following the death of a family member. He acknowledged that he struggled to store the medication at the required temperature while traveling and was often late in administering injections. The decision was made to terminate therapy at week 23.

When bulevirtide became reimbursed in England in June 2023,^12^ the decision was made to try again with therapy. Bulevirtide injections were started again and were largely well tolerated. There was an excellent and sustained biochemical and virological response (Table 4), and the patient continues on the treatment at the time of writing.

## Discussion

As shown in the above patient cases, with appropriate support, patients with HDV can achieve positive results with bulevirtide therapy, demonstrating a reduced viral load and improvements in key measures of hepatic function—regardless of background or social circumstances. Challenging social circumstances should not be seen as insurmountable barriers to care, nor as factors that will determine a patient’s adherence, as management strategies can be tailored to meet the specific needs and requirements of individual patients.

Providing convenient care that is tailored to the individual needs of the patient has been demonstrated to help with treatment adherence across disease states.^21^^,^^22^ For patients prescribed bulevirtide, this may involve initiating therapy at a time that accommodates their personal circumstances, or working with third-party services to provide practical support that addresses medication storage and logistical challenges.

Where possible, initiation of bulevirtide should not coincide with disruptive life events and should be at a time when the patient is emotionally stable and able to commit to long-term treatment. It is also important to consider how patients will travel with bulevirtide, especially in areas with more limited resources where appropriate storage may be a challenge. Treatment may be paused should an individual’s circumstances change, though the patient should be closely monitored during time off treatment given the potential for hepatitis exacerbation after bulevirtide discontinuation.^11^ It is also encouraging to note that, per patient case 4, treatment with bulevirtide can be tried again successfully, even if there have been issues with the first attempt. Regular check-in appointments with patients receiving bulevirtide can help to identify any changes to their circumstances that may impact their ability to adhere to treatment, as well as providing an opportunity to monitor their response to therapy and their injection technique.

Maintaining long-term engagement with care can be challenging in some patient groups, including intravenous drug users. Meeting patients in familiar or trusted environments, such as the drug service clinic used by patient 3, can further support (re-)engagement with hepatitis care and help to foster stronger patient–HCP relationships. Utilizing outpatient treatment centers, such as the OPAT service used by patient 2, can help to facilitate safe and confidential administration of bulevirtide for individuals whose housing or living arrangements may otherwise impede their access to care.

Nonadherence is a widespread issue, affecting patients across all social and demographic backgrounds; all patients should be offered equitable, confidential access to care, regardless of background or circumstances.^22^^,^^23^ HCPs should avoid letting biases influence patient interactions, as building a strong, trusting patient–HCP relationship is fundamental to promoting adherence.^14^^,^^24^^,^^25^ Equally, education and awareness campaigns can help empower patients to take a more active role in their care, as a greater understanding of the disease and the implications of treatment has consistently been shown to support adherence.^14^^,^^22^^,^^24^^,^^25^ Such initiatives should account for cultural differences, as well as address language and literacy barriers through the use of interpreters or translated materials, to ensure that education is accessible for all.^23^^,^^26^

It is worth acknowledging that giving due consideration to understand individual patient circumstances requires time that is already lacking for many HCPs. Therefore, a multidisciplinary approach is key, with roles determined according to the capacity and requirements of individual centers, and strong communication between HCPs of different departments. Developing robust treatment pathways that integrate pharmacy- or nurse-led teams to increase clinic capacity could help improve treatment access while sharing the workload.^27^

Despite advances in the management of HDV infection, several unmet needs remain. Limitations in diagnostic assays, gaps in systematic screening, and low awareness of HDV among both the public and HCPs continue to delay diagnosis for many patients.^1^ Care can be further hindered by stigma and the spread of misinformation regarding hepatitis, with patients often unclear on their disease prognosis, transmission risk, and the potential benefits of treatment.^15^^,^^28^^,^^29^ Targeted education and supportive, non-judgmental care are essential to support patients throughout their treatment journey.

The approval and reimbursement of bulevirtide as the first licensed treatment for HDV in the United Kingdom^9^^,^^12^ marked a positive step for the management of HDV, yet access to such treatment is limited to specialist hospitals and under strict clinical criteria.^27^ Furthermore, the optimal duration for bulevirtide treatment is yet to be established.^10^ Recent trial evidence has shown that longer treatment may be most beneficial, with virologic and biochemical responses to bulevirtide maintained or improved from week 48 through to week 96.^30^ Current guidance states that treatment should be continued for as long as associated with a clinical benefit; discontinuation may be considered in the case of sustained (6 months) hepatitis B surface antigen seroconversion or loss of virological and biochemical response.^10^^,^^11^

HDV predominantly affects vulnerable populations, with the additional social challenges faced by this group exacerbating health inequalities.^7^^,^^16^ Vulnerable populations most at risk of HDV, such as migrants and asylum seekers, can have impaired access to healthcare on a systematic level, with many migrants facing challenges accessing healthcare in their new country.^7^^,^^31^ In the United Kingdom, many services are unaware of the rights of migrants regarding access to the NHS, with many patients turned away and denied access to care.^32^ It is also important to note that the patient cases reported here are from UK NHS hospitals; in countries that lack universal healthcare, insurance and cost of treatment represent a significant barrier to patients receiving care.^14^

## Conclusion

The patient cases presented in this series highlight that patients with HDV can achieve positive results with bulevirtide therapy, regardless of social backgrounds. Our experiences support the value of a flexible, patient-centered treatment approach to care and challenge the preconceived assumptions that certain social circumstances are insurmountable barriers to treatment adherence. Further work is needed to determine the most effective support strategies and treatment referral pathways that can be implemented within existing healthcare capacity constraints. Providing HCPs with the tools and training needed to deliver patient-centered, equitable HDV management, alongside enhanced patient education and targeted efforts to reduce stigma and misinformation, will be key to optimizing outcomes and reducing disparities for individuals living with HDV infection. By shifting the perception that social circumstances are prohibitive to adherence, we can promote a more inclusive approach to initiating bulevirtide therapy, ultimately improving health outcomes for patients with HDV.
